# Emotional salience but not valence impacts anterior cingulate cortex conflict processing

**DOI:** 10.3758/s13415-022-01025-9

**Published:** 2022-07-25

**Authors:** Suvarnalata Xanthate Duggirala, Michel Belyk, Michael Schwartze, Philipp Kanske, Sonja A. Kotz

**Affiliations:** 1 Department of Neuropsychology and Psychopharmacology, Faculty of Psychology and Neuroscience, Maastricht University, 6229 ER Maastricht, Netherlands; 2 Faculty of Psychology, University of Lisbon, Lisbon, Portugal; 3 Department of Psychiatry and Neuropsychology, School of Mental Health and Neuroscience, Maastricht University Medical Center, Maastricht, Netherlands; 4 Department of Speech, Hearing, and Phonetic Sciences, Faculty of Psychology and Language, University College London, London, UK; 5 Department of Psychology, Edge Hill University, Ormskirk, UK; 6 Faculty of Psychology, Technische Universität Dresden, Dresden, Germany

**Keywords:** Emotion, Salience, Valence, Conflict processing, fMRI

## Abstract

Stimuli that evoke emotions are salient, draw attentional resources, and facilitate situationally appropriate behavior in complex or conflicting environments. However, negative and positive emotions may motivate different response strategies. For example, a threatening stimulus might evoke avoidant behavior, whereas a positive stimulus may prompt approaching behavior. Therefore, emotional stimuli might either elicit differential behavioral responses when a conflict arises or simply mark salience. The present study used functional magnetic resonance imaging to investigate valence-specific emotion effects on attentional control in conflict processing by employing an adapted flanker task with neutral, negative, and positive stimuli. Slower responses were observed for incongruent than congruent trials. Neural activity in the dorsal anterior cingulate cortex was associated with conflict processing regardless of emotional stimulus quality. These findings confirm that both negative and positive emotional stimuli mark salience in both low (congruent) and high (incongruent) conflict scenarios. Regardless of the conflict level, emotional stimuli deployed greater attentional resources in goal directed behavior.

Navigating a complex environment requires the selection of appropriate responses while ignoring conflicting information. This implies that efficient attentional control is required to resolve conflict that may arise from opposite action tendencies triggered by both relevant and irrelevant stimuli (Norman & Shallice, 1986, 2000; Posner & Fan, 2008). Such situations are modeled in experimental settings using conflict paradigms where irrelevant distractors surround task-relevant targets, thereby creating either matching (congruent) or mismatching (incongruent) action tendencies (Eriksen & Eriksen, 1974; Simon & Rudell, 1967; Stroop, 1992). Prolonged response times (RT), increased error rates, and elevated neural activity in the anterior cingulate cortex (ACC) are typically associated with incongruent relative to congruent trials (Barch et al., 2001; Fan et al., 2003, 2008; Kerns, 2006; Kerns et al., 2005; van Veen et al., 2004). These factors suggest high attentional control in conflict processing. Additional attentional control processes and systems are activated by salient situations often signaled by emotional stimuli (Norman & Shallice, 1986, 2000). For example, a potentially threatening situation, such as encountering a snake, may pose a challenge to well-being and invoke avoidance behavior in the form of freeze, flight, or fight responses. Emotionally evocative stimuli can modulate attentional control in conflict processing by slowing down or speeding up cognitive and behavioral responses. However, negative and positive emotional stimuli motivate different response strategies, because they are associated with avoidance and approach behavior, respectively (Fredrickson, 1998, 2001; Fredrickson & Branigan, 2005). These stimuli therefore may either stimulate different approaches to attentional control in conflict processing or merely act as salience markers by triggering attention. Research has focused more on negative emotions and interpreted them as evolutionary considerations as a threatening stimulus can affect survival and well-being. However, there is little evidence whether valence influences attentional control in a specific way in conflict processing both in behavioral and neural terms.

Task-irrelevant emotional information can either be presented before a conflict trial or concurrent with the task relevant stimulus dimension such that it is present continuously throughout the conflict trial (for details on emotional manipulation in the cognitive control tasks see review Duggirala et al., 2020). Prior evidence on emotional priming reports disrupted continuous maintenance of contextual information and challenges ideas of attentional reorientation. Negative and positive emotional priming have either resulted in slower responses (Blair et al., 2007; Hart et al., 2010; Melcher et al., 2011; Padmala et al., 2011; Straub et al., 2020) or have shown no emotional interference compared with neutral priming in conflict processing tasks (Cohen et al., 2011; Cohen & Henik, 2012). On the other hand, continued passive exposure to emotional information competes with task-relevant processes for attentional resources in conflict processing and ultimately influences task-performance. Studies from this latter category where (task-irrelevant) emotion is part of the stimulus dimension have shown more varied results.

Studies using modified versions of flanker or Simon tasks with stimuli connoting a negative emotion have reported facilitated conflict processing, with faster reaction times for negative incongruent than neutral trials (Kanske & Kotz, 2011c, 2012b; Zinchenko et al., 2015). Conversely, several studies using emotional Stroop tasks with negative stimuli described inhibition of conflict processing and correspondingly longer color-naming latencies for negative than neutral trials (Ben-Haim et al., 2014; Brennan et al., 2015; Frings et al., 2010; Frings & Wuhr, 2012; Malhi et al., 2005; Mitterschiffthaler et al., 2008; Mohanty et al., 2005; Rahm et al., 2013; Veroude et al., 2013; Wingenfeld et al., 2009). Although emotion is task-irrelevant per se, it is a behaviorally relevant stimulus dimension in these tasks. Inconsistent behavioral findings between these tasks might be attributed to context, the degree of interference created by stimuli, and the corresponding strength of the resulting conflict. For example, although in the emotional Stroop task, interference is produced by the emotional meaning of a word, an additional layer of interference is created by the flanker colors in the flanker task. A stimulus connoting a negative emotion may be distracting when conflict is low (e.g., color-word emotional Stroop trial) and results in slower responses (Ben-Haim et al., 2014; Brennan et al., 2015; Malhi et al., 2005; Mitterschiffthaler et al., 2008; Mohanty et al., 2005; Rahm et al., 2013; Veroude et al., 2013; Wingenfeld et al., 2009). Conversely, the same stimulus in a high-conflict context (e.g., incongruent flanker or Simon trials) may recruit additional resources and activate neural networks to cope with the prospect of increased threat (Holtz et al., 2012), leading to faster responses.

Positive emotions have a different ethological role. Rather than a narrowed focus on immediate and pressing reactions, they motivate a broadening and expansion of attentional focus when circumstances are favorable (Fredrickson, 1998, 2001; Fredrickson & Branigan, 2005). However, the exact mechanisms underlying the effect of positive stimuli on attentional control in conflict processing remain unclear. Like negative emotions, positive emotions facilitate conflict processing in flanker and Simon tasks and lead to shorter reaction times in incongruent positive than neutral trials (Kanske & Kotz, 2011a, 2011d; Xue et al., 2013). Similarly and consistent with negative emotions, emotional Stroop tasks using positive words yield inhibition of conflict processing and longer reaction times (Dresler et al., 2009). However, some studies using modified versions of the flanker task with positive verbal or audio-visual stimuli also showed no difference in reaction times compared with negative (Li et al., 2014) or neutral trials (Wu & Zhang, 2019; Zinchenko et al., 2017). Studies using emotional Stroop tasks with positive words likewise reported no difference in reaction times over negative or neutral trials (Malhi et al., 2005; Richards et al., 1992). The influence of positive emotion on attentional control in conflict processing thus remains unclear.

Although different conflict processing tasks might engage distinct sub-processes of attentional control to regulate emotional interference, they might share a similar neural basis. Increased ACC activation is a typical finding in most conflict paradigms, including Stroop, flanker, and Simon tasks (Fan et al., 2003). However, studies using emotionally evocative stimuli in these tasks further report a bifurcation in the functionality of dorsal and ventral parts of the ACC (Kanske & Kotz, 2011b, 2011c; Kim et al., 2011; Milham & Banich, 2005; Weissman et al., 2005). The dorsal portion of the ACC is associated with conflict processing independent of emotional stimulus quality (Kanske & Kotz, 2011b, 2011c; Xu et al., 2016). However, the ventral part of the ACC is sensitive to emotional conflict and to the resolution of conflict by emotional distractors (Etkin et al., 2006; Kanske & Kotz, 2011b, 2011c). These findings were obtained with negative emotional stimuli. The sensitivity of these regions to positive emotional stimuli in conflict processing is therefore still unknown. Studies that compared positive to negative emotional stimuli within a modified version of emotional Stroop task, did not report any significant brain activity in ACC (Arioli et al., 2021). A similar picture emerges with regards to neural networks. Meta-analyses of neuroimaging studies as well as individual neuroimaging studies using irrelevant emotional stimuli in conflict tasks report increased activity in a fronto-parietal-temporal network consisting of the ACC, inferior, middle and medial/superior frontal gyrus, the dorsolateral prefrontal cortex, the inferior and superior parietal lobule, the angular and supramarginal gyrus, the orbitofrontal cortex, the insula, the inferior and superior temporal gyrus, the precuneus, the precentral and postcentral gyrus and amygdala (Cromheeke & Mueller, 2014; Malhi et al., 2005; Mohanty et al., 2005; Rahm et al., 2013; Song et al., 2017; Veroude et al., 2013; Wingenfeld et al., 2009). Furthermore, experiments reporting enhanced task-performance (faster RTs/low errors) observed increased activity in the inferior and superior frontal gyrus and the angular gyrus, whereas diminished performance was linked to increased activation in the medial/superior frontal gyrus, the precuneus, the inferior frontal gyrus, the amygdala and the fusiform gyrus (Cromheeke & Mueller, 2014). Most of the studies included in these meta-analyses (Cromheeke & Mueller, 2014; Song et al., 2017) compared negative to neutral stimuli, neglecting positive emotions. There is a clear void in the literature regarding the role of positive emotion on conflict processing.

The current study extends prior work (Kanske & Kotz, 2011c) to test whether (i) negative and positive emotions have similar or opposing effects on task performance in conflict processing, (ii) the dACC and vACC activate differently for negative and positive emotional stimuli in conflict processing, and (iii) negative and positive emotions engage distinct neural systems in conflict processing. To answer these questions, we employed a pre-validated (Kanske & Kotz, 2011c) verbal adaptation of the Eriksen Flanker task with standardized neutral, negative, and positive German words during functional magnetic resonance imaging (fMRI). We hypothesized that stimuli connoting a negative emotion would activate the neural systems involved in goal-driven processes facilitating conflict processing whereas stimuli with a positive connotation might engage systems involved in reward and memory retrieval leading to distraction and inhibition of conflict processing.

## Methods

### Participants

Twenty-three healthy adults participated in the study. A priori power calculations using G-Power statistical software (Faul et al., 2007) indicated that with α = 0.05 and power (1-error probability) = 0.85 and a medium effect size of 0.25, a sample of a minimum 21 participants would be required for the current task design. This sample size also is supported by previous publications using a similar task design (Kanske & Kotz, 2011b, 2011c, 2012b). Two participants were excluded from further analyses (one due to technical issues during data acquisition and the other for revealing the exclusion criteria [left-handedness] after the experiment), leaving a final sample of 21 right-handed healthy adults (9 females; age range: 19-26 years; mean age = 22.29, SD = 1.95 years). All participants were native German speakers and had normal or corrected-to-normal vision at the time of the experiment. Participants reported to be healthy and had no history of neurological or psychiatric disorders. The study was approved by the ethical review committee psychology and neuroscience, Maastricht, The Netherlands (ERCPN- 176_01_02_2016_A1). All participants provided their informed consent before the start of the study. They either received financial compensation or study credits for taking part in the study.

### Experimental paradigm and stimuli

**Fig. 1:**
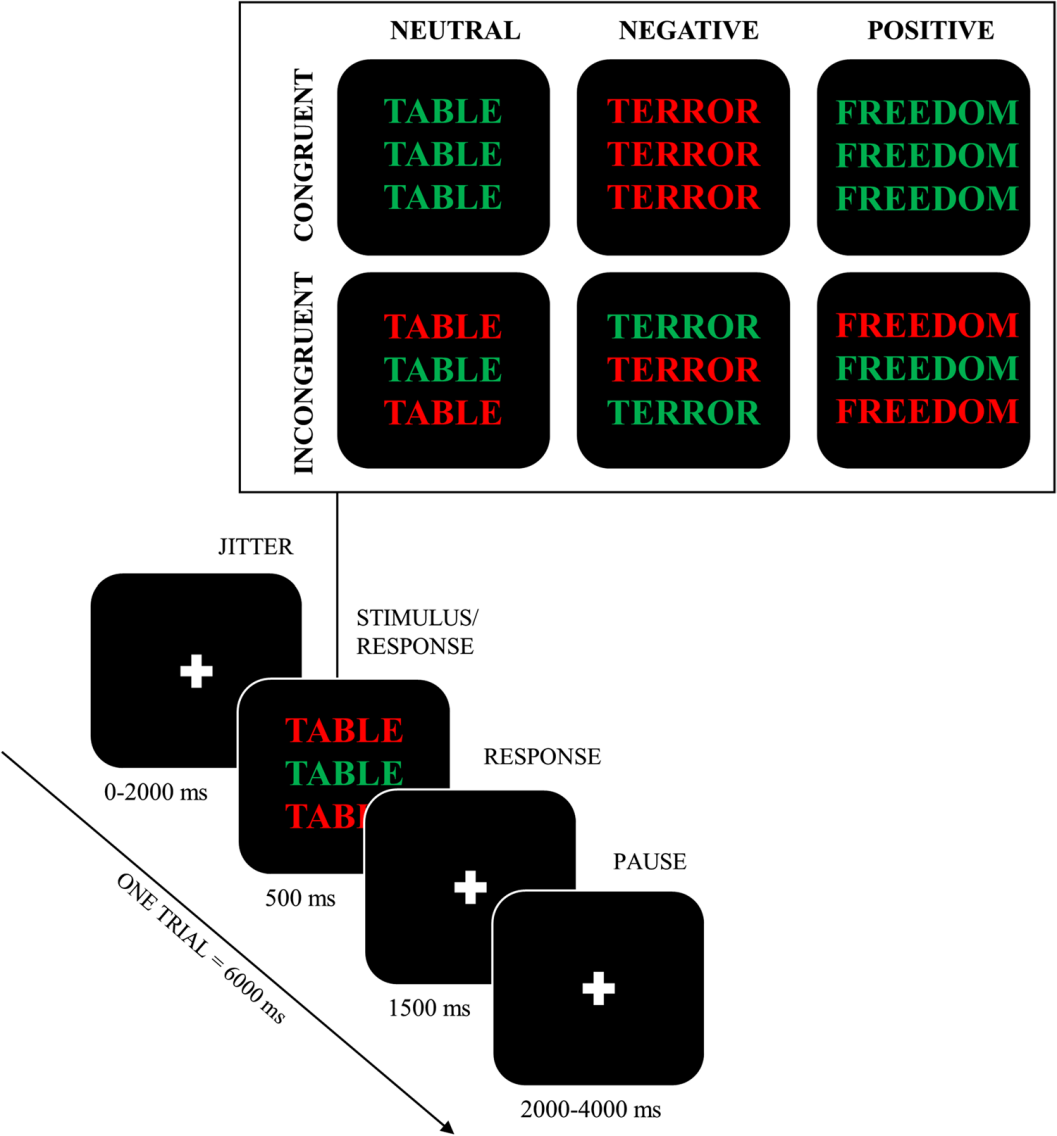
Modified version of the flanker task (Kanske & Kotz, 2011c)

A modified verbal version of the visual flanker task was employed in the fMRI scanner. Participants were asked to identify the display color of a centrally presented word using their right index finger and right middle finger, while ignoring the color of two flanker words positioned above and below the target word (Fig. 1) (Kanske & Kotz, 2011c). Flanker and target word colors could be identical or different, creating congruent (C) and incongruent (IC) trials. Forty pre-standardized German nouns belonging to neutral, negative, and positive emotional categories, respectively, were selected from a corpus that had been validated for emotional valence (negative-neutral-positive), arousal (low-high), and concreteness (concrete-abstract) (Kanske & Kotz, 2010). These word groups significantly differed in valence and arousal (Table 1) (Kanske & Kotz, 2010, 2012a). Accordingly, emotion was task-irrelevant but part of the behaviorally relevant stimulus dimension.

**Table 1:** Mean reaction times and percent correct responses

Category	Congruent		Incongruent		Conflict effect
	RT (ms)	% Correct responses	RT (ms)	% Correct responses	
Neutral	628.76 (83.71)	96.19 (5.22)	643.26 (88.91)	96.43(4.22)	14.49
Negative	632.52 (88.29)	96.90 (4.32)	642.44 (88.41)	95.95 (5.15)	9.91
Positive	619.25 (80.70)	96.67 (5.08)	645.02 (84.93)	92.85 (8.74)	25.77

The stimuli were presented in a fully randomized event-related design comprising two identical runs consisting of 80 trials each. These trials were equally distributed among null, neutral, positive, and negative categories. Within each category (i.e., neutral, negative, and positive), there were ten congruent and incongruent trials, respectively (Kanske et al., 2011; Kanske et al., 2013). Each trial lasted for 6 seconds. Within each trial, a stimulus was displayed for 500 ms followed by a response time of 1,500 ms. Stimulus onset within a trial was jittered between 0 to 2,000 ms at 113-, 563-, 1,013-, 1,463-, 1,913-ms intervals to avoid temporal orienting or habituation effects (Fig. 1). Each word stimulus was used once, and there were no repetitions during the task. The task therefore comprised six conditions in total (incongruent neutral, congruent neutral, incongruent negative, congruent negative, incongruent positive, congruent positive), each consisting of 20 trials. A fixation cross was displayed during the null trials. Response mapping and the order of the runs were counterbalanced across participants.

### Procedure

The study took place in a single session, comprising two parts. In the first part, participants filled in an online questionnaire that recorded demographic information. Participants were then familiarized with the task outside the scanner to avoid training effects inside the scanner. The task was programmed and presented using Presentation software (Neurobehavioral Systems, Inc., Version 18). In the second part, participants underwent anatomical and functional scanning. Stimuli were rear-projected onto a screen with black background (Eiki LCD projector, 60 Hz refresh rate, 1,024 × 768 display resolution), which was visible for the participants through a mirror attached to the head-coil. Participants gave their responses via an MRI-compatible response keypad.

### MRI data acquisition

Participants were scanned at a Siemens 3-T MRI scanner as they lay in headfirst supine position with their head movement restricted by foam cushions. Following a localizer sequence, high resolution anatomical images were acquired via a T1-weighted MPRAGE sequence (TR = 2,250 ms, TE = 2.21 ms, FoV = 256 mm, flip angle = 9°, slice thickness = 1 mm, number of slices = 192, orientation = sagittal, voxel size = 1 mm^3^ isotropic). Anatomical scans were followed by a short reversed phased gradient echo-planar imaging (EPI) scan, after which normal phased functional scans were taken during which participants performed the modified version of the Ericksen flanker task. A T2*-weighted EPI sequence was used to acquire blood oxygen level dependent sensitive functional images (TR = 2,000 ms, TE = 30 ms, flip angle = 70°, number of slices = 60 axial slices, slice thickness = 2 mm, interslice gap = 1 mm, FoV = 208 mm, in-plane resolution = 2 mm × 2 mm, acquisition = interleaved ascending).

### Analyses

#### Behavioral data

Performance accuracy and mean RTs were calculated as % hits and mean response times to a stimulus, respectively. Error trials and trials with RT longer than 1500 ms were excluded from further data analysis. Conflict scores were calculated by subtracting mean RT for congruent trials from mean RT for incongruent trials. A 2 × 3 repeated measures ANOVA was performed on mean RT and performance accuracy scores to reveal the main effect of congruency and emotion, and their interaction. Statistical package for social sciences (SPSS, version 18.0, Chicago, IL) was used to analyze the behavioral data.

#### Neuroimaging data

The functional images were preprocessed and analyzed using SPM12 (Wellcome Department of Neurology, Institute of Neurology, London, UK), implemented in MATLAB 2016 (Mathworks Inc., Sherborn, MA). Before preprocessing, distortion correction was performed using the TOPUP algorithm, which estimates image distortions by comparing EPI data collected with normal and reversed phase encoding directions to remove artifacts caused by magnetic susceptibility (Jenkinson et al., 2012). Preprocessing steps involved correcting for differences in slice time using the middle slice as the reference. A mean image of all slice time corrected functional scans of each participant was created, to which individual volumes were spatially realigned using rigid body transformation. Head movements in all three dimensions were within the 2-mm threshold. Structural images of each participant were co-registered with their mean functional image and all functional images were normalized to the Montreal Neurological Institute (Montreal, Quebec, Canada) T1 template. Then, the images were spatially smoothed using an 8-mm, full-width at half maximum Gaussian filter. Further statistical analyses were performed on each participant’s data using the general linear model (GLM). The design matrix consisted of two sessions corresponding to each run. In each run, 7 regressors corresponding to baseline (null) and active conditions (congruent and incongruent regressors for neutral, positive, and negative emotion, respectively) were defined. For these regressors, the onset of the stimulus represented the event onset. The jittered fixation cross presented before or after were not included in the modeling. This was done because we already have a baseline (null) condition in the design, which was modeled in the GLM. Trials corresponding to wrong response (errors) also were excluded from the modeling to keep the fMRI analysis comparable to the behavioral analysis. In addition, six motion regressors derived from the rigid body realignment were included to model linear residual movement effects.

At the participant level, contrasts corresponding to incongruent neutral, incongruent negative, incongruent positive, congruent neutral, congruent negative, and congruent positive conditions were defined. At group level, these contrasts from each participant were transferred to random effects analysis. A 2 × 3 within subjects repeated measures ANOVA with congruency (2 levels) and emotion (3 levels) as main factors was performed using a simple flexible factorial model in SPM.

### ROI analysis

Functional search volumes were defined by drawing spheres of 10-mm radius around the peak MNI coordinates drawn from an independent sample (Kanske & Kotz, 2011c) defining the dorsal and ventral ACC. Parameter estimates were extracted from the beta images by defining a sphere of 6 mm around a single participant peak within the functional search volume. Effect sizes were calculated as percent signal change (PSC = [beta(task)*max(HRF)*100]/[beta(constant)] where beta(task) refers to the parameter estimate of the effect of interest, max(HRF) is the maximum of the a single event of the current duration convolved with the current basis function, and beta(constant) the parameter estimate of the current session constant) using the rfx plot toolbox (http://rfxplot.sourceforge.net/) with task (all regressors correspond to active conditions, i.e., incongruent neutral, congruent neutral, incongruent negative, congruent negative, incongruent positive, congruent positive) versus null contrast as the unbiased effect of interest (Gläscher, 2009). PSC estimates the evoked change in BOLD response for a condition between two conditions. For more detailed information on this procedure, please refer to http://rfxplot.sourceforge.net/documentation/manual.pdf.

### Whole brain analysis

Whole brain activations corresponding to the following contrasts were assessed: (i) main effect of congruency (incongruent > congruent), (ii) main effect of emotion ([negative + positive] > neutral), (ii) interaction of congruence and emotion, (iv) main effect of negative emotion (negative > neutral), and (v) main effect of positive emotion (positive > neutral). Final whole brain activations are reported at *p* < 0.001 and a minimum cluster size of 17 contiguous voxels. We applied a well-validated Monte-Carlo simulations approach to correct for multiple comparisons (cluster_threshold_beta.m; The Mathworks, Natick, MA; 2015a; Slotnick et al., 2003; Slotnick & Schacter, 2006; Slotnick, 2017a, 2017b). After running 10,000 simulations, it was determined that for an individual voxel threshold of *p* < 0.001, a cluster-extent threshold of 17 contiguous voxels (equivalent to a volume of 136 mm^3^) was necessary to correct for multiple comparisons to achieve a significance level of *p* < 0.05. Therefore, only clusters of activation equal or exceeding that size were considered significantly active.

## Results

### Behavioral data

**Fig. 2:**
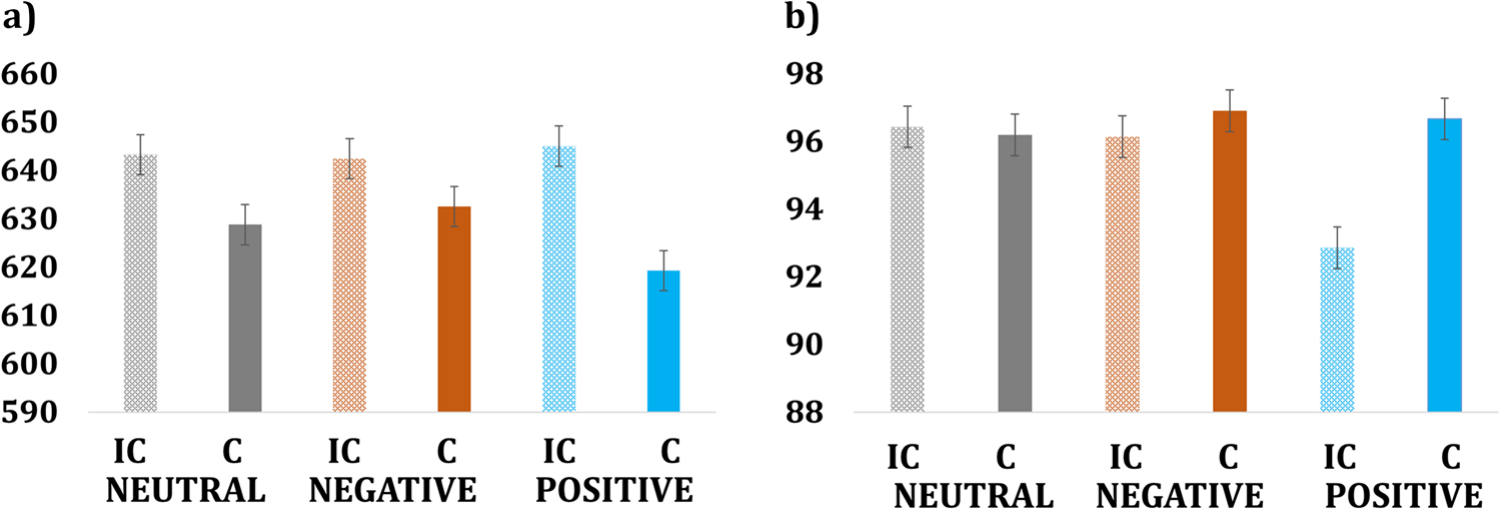
**a** Mean reaction times for correct responses in milliseconds. **b** Mean accuracy expressed as percent correct responses for each condition. Vertical bars indicate the standard error of mean. IC = incongruent, C = congruent; *p < 0.05; **p < 0.01; ***p < 0.001

The within-subjects ANOVA of mean RTs with the factors congruency (incongruent and congruent) and emotion (neutral, negative, and positive) yielded a significant main effect of congruency (F(1, 20) = 16.081, *p* = 0.001; ${\eta}_p^2$= 0.446), whereas there was no significant effect of emotion (F(2, 40) = 0.278, *p* = 0.759; ${\eta}_p^2$ = 0.014) or a congruence-by-emotion interaction (F(2, 40) = 1.013, *p* = 0.372; ${\eta}_p^2$ = 0.048) (Fig. 2a; Table 1). Analysis of mean percent accuracies yielded no significant main effects of congruency (F(1, 20) = 3.828, *p* = 0.065; ${\eta}_p^2$ = 0.161), emotion (F(2, 40) = 2.068, *p* = 0.14; ${\eta}_p^2$ = 0.094) or interaction of these factors (F(2, 40) = 2.270, *p* = 0.117; ${\eta}_p^2$= 0.102) (Fig. 2b; Table 1).

### Neuroimaging data

#### ROI analyses

**Fig. 3:**
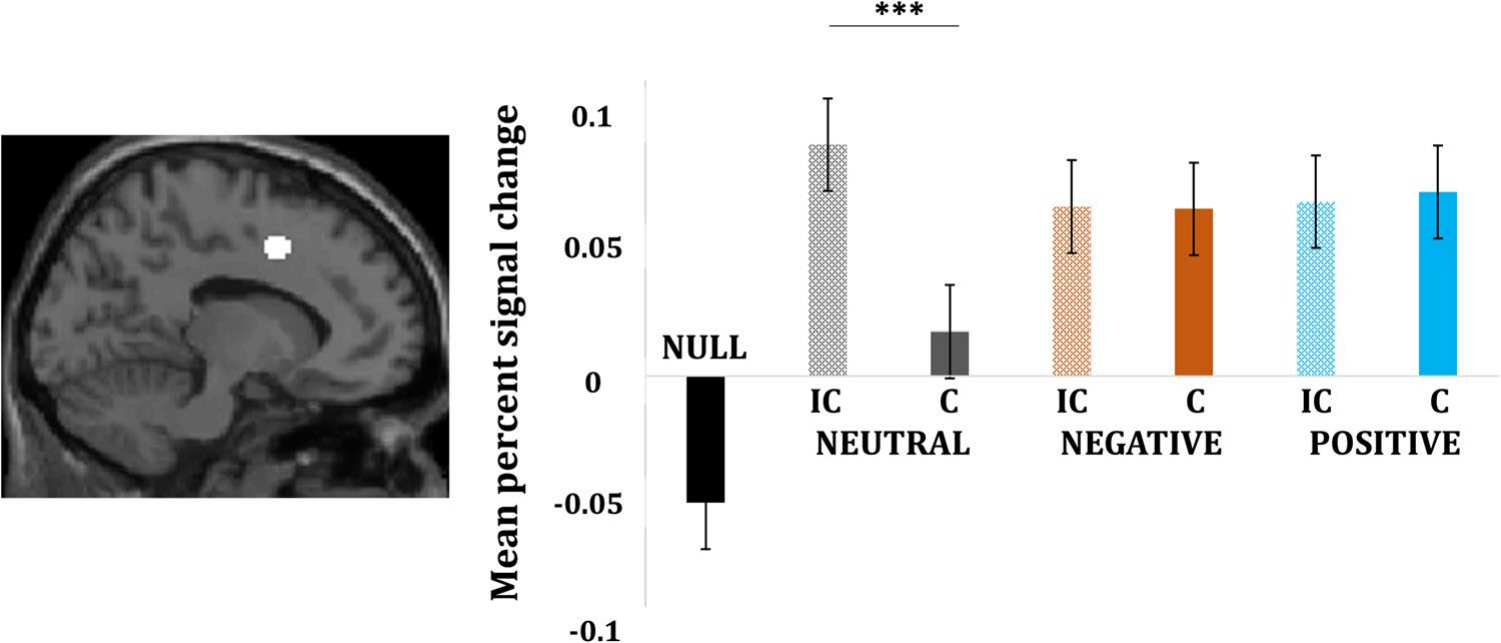
Percent signal change and contrast estimates for dorsal ACC [−11 7 44]. Vertical bars in the graphs indicate SEM. Activations are reported at *p* < 0.001, cluster FDR < 0.05 (in accordance with Monte Carlo simulations), cluster size >17 voxels; IC = incongruent, C = congruent. Activations are reported at *p* < 0.001, cluster FDR < 0.05 (in accordance with Monte Carlo simulations), cluster size >17 voxels; IC = incongruent; C = congruent; *p < 0.05; **p < 0.01; ***p < 0.001

Repeated-measures ANOVA were performed on percent signal change (PSC) values with congruency and emotion as factors for each ROI (Fig. 3). The dorsal ACC showed no significant main effect of congruency ((F(1, 20) = 3.582, *p* = 0.07; ${\eta}_p^2$ = 0.152), no significant effect of emotion (F(1, 20) = 0.844, *p* = 0.44; ${\eta}_p^2$ = 0.04), but a significant congruence-by-emotion interaction (F(2, 40) = 8.458, *p* = 0.001; ${\eta}_p^2$ = 0.30). Follow-up analyses revealed a significant difference between incongruent compared with congruent neutral (t(20) = 4.722, *p* < 0.001) but not for incongruent vs. congruent negative (t(20) = −0.095, *p* = 0.92) or incongruent vs. congruent positive trials (t(20) = −1.009, *p* = 0.32). No significant neural activity was found in the ventral ACC.

#### Whole brain analyses

**Fig. 4:**
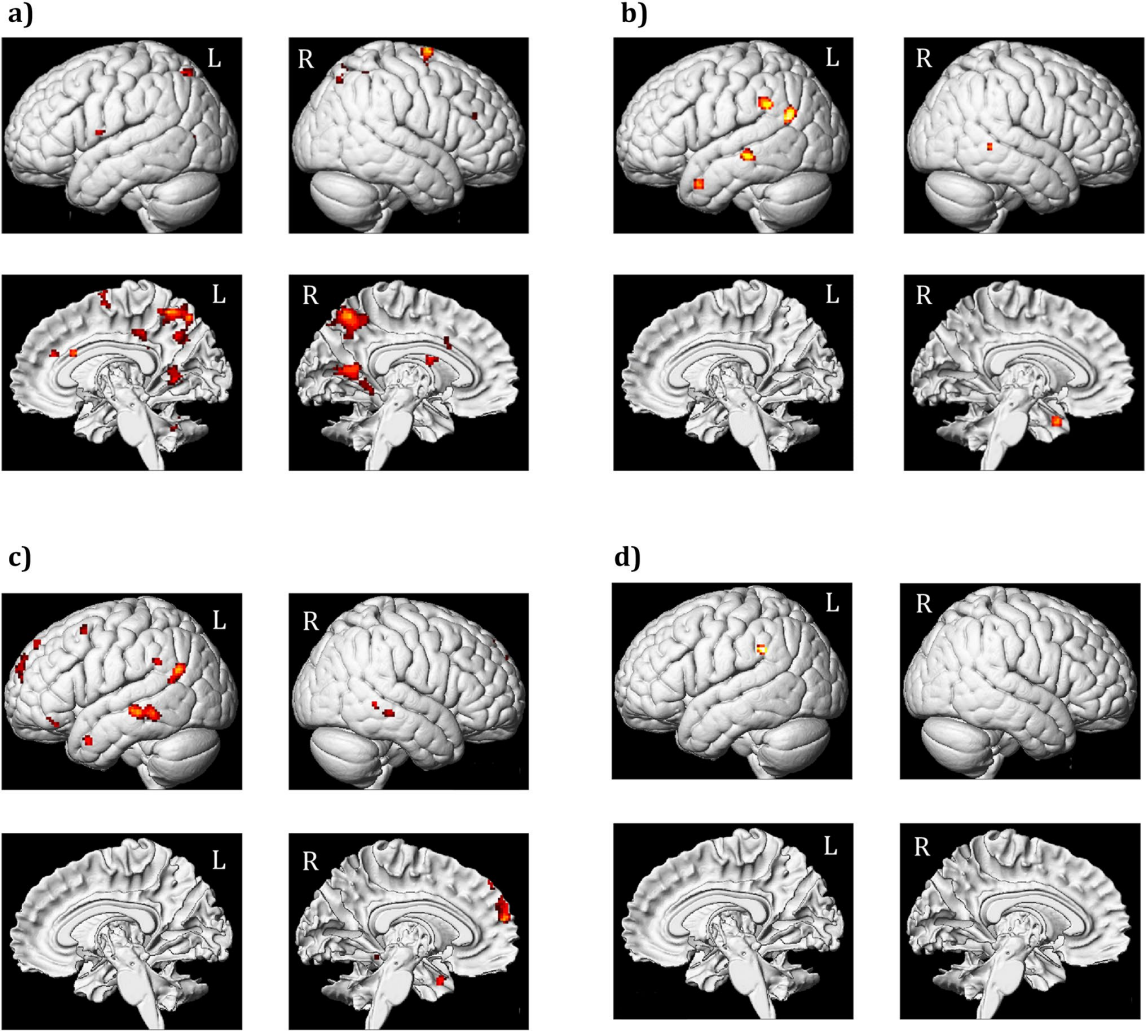
Whole brain activations: **a** Main effect of congruency *(IC > C irrespective of stimulus type)*; **b** Main effect of emotions [(Positive + Negative) vs. Neutral]; **c** Main effect of negative (vs. neutral) emotion; **d** Main effect of positive (vs. neutral) emotion. *Note:* Activations are reported at *p* < 0.001, cluster FDR < 0.05 (in accordance with Monte Carlo simulations), cluster size >17 voxels; IC = incongruent, C = congruent; *p < 0.05; **p < 0.01; ***p < 0.001

A 2 × 3 within subjects ANOVA with congruency and emotion as main factors using a simple flexible factorial model in SPM yielded a significant main effect of congruency in a frontoparietal network consisting of the right superior frontal gyrus, the left and the right superior parietal lobule, the right precuneus, the right middle frontal gyrus, the left middle cingulate cortex, the left precentral gyrus, and the right cerebellum (Fig. 4; Table 2). The main effect of emotion was found in the left middle temporal pole, left middle temporal gyrus, left supramarginal gyrus, left angular gyrus and right middle temporal gyrus. The main effect of emotion was further broken down into the main effect of negative and positive emotion (Table 2). Last, an interaction of congruence and emotion did not confirm significant whole brain activity.

**Table 2:** Whole brain activations for within subjects ANOVA with congruency and emotions as factors. All activations are reported at *p* < 0.001, cluster FDR < 0.05 (in accordance with Monte Carlo simulations), cluster size > 17 voxels

Brain region	BA	Peak MNI coordinate			Voxel level	Cluster size
		x (mm)	y (mm)	z (mm)	T score	K (voxels)
a) Main effect of congruency (IC > C irrespective of the stimulus type)						
R. Superior frontal gyrus	-	16	0	76	4.46	129
R. Superior frontal gyrus*	6	22	-6	62	3.44	
L. Superior parietal lobule	-	-20	-64	52	4.42	341
R. Precuneus	-	14	-56	52	4.00	331
R. Superior parietal lobule*	7	22	-70	50	3.71	
R. Middle frontal gyrus	-	32	34	24	3.64	25
L. Precentral gyrus	-	-52	0	12	3.59	19
L. Middle/anterior cingulum	32	-8	14	36	3.47	22
R. Cerebellum_6	-	28	-60	-28	3.46	53
b) Main effect of emotion (emotional > neutral trials irrespective of the congruency)						
L. Middle temporal pole	-	-44	8	-28	4.12	46
L. Middle temporal gyrus	-	-54	-28	-6	4.06	95
L. Supramarginal gyrus	40	-60	-44	32	3.91	92
L. Angular gyrus	39	-50	-62	26	3.88	114
R. Middle temporal gyrus	-	56	-42	0	3.42	17
c) Main effect of negative emotion (negative > neutral trials)						
L. Middle temporal gyrus	-	-56	-28	-6	4.39	250
L. Middle temporal gyrus*	21	-64	-38	-6	3.71	
L. Middle temporal gyrus*	-	-58	-44	-10	3.48	
L. Middle temporal pole	38	-42	8	-30	4.05	36
L. Superior frontal (medial)	10	-6	60	22	3.80	200
L. Superior frontal (medial)*	9	-4	54	28	3.58	
R. Middle temporal gyrus	-	52	-32	-8	3.79	38
L. Angular gyrus	39	-52	-62	26	3.76	132
L. Superior temporal gyrus *	22	-62	-58	18	3.48	
L. Middle frontal gyrus	-	-40	12	58	3.64	22
L. Superior frontal (medial)	6	-10	48	46	3.58	26
L. Supramarginal gyrus	40	-60	-46	32	3.49	28
R. Middle temporal gyrus	-	56	-42	-2	3.44	17
L. Inferior frontal (orbital) gyrus	-	-40	34	-18	3.36	21
L. Inferior frontal (orbital) gyrus*	11	-44	38	-12	3.24	
d) Main effect of positive emotion (positive > neutral trials)						
L. Supramarginal gyrus	40	-60	-42	32	3.47	34

*Subpeak within a cluster. *L* = left, *R* = Right

## Discussion

The current study examined whether negative and positive emotions differentially modulate attentional control in conflict processing or act as global indicators of salience in conflict processing. In particular, the study sought to examine the valence specific influence of emotion on dorsal and ventral ACC in conflict processing. The behavioral results replicated the classic conflict effect, i.e., slower responses for incongruent than congruent trials (Eriksen & Eriksen, 1974). However, the task did not produce a significant effect of emotional valence on conflict processing. Region of interest analysis revealed a general role of the dorsal ACC in monitoring conflict and appropriate response selection irrespective of emotional valence or the level of conflict (Fig. 3). Furthermore, whole brain analyses showed that both negative and positive emotion elicited activity in an extensive network of brain regions associated with controlling the response to interference caused by emotion in conflict processing. This might indicate that emotion marks salience and engages attentional control in order to maintain task-performance.

### Behavioral response conflict

We replicated the behavioral main effect of conflict (Eriksen & Eriksen, 1974) such that slower responses for incongruent as compared to congruent trials were observed. However, we did not find any significant effect of emotional valence on conflict processing. In this respect, our findings differ from previous behavioral results obtained with a similar version of the flanker task with negative emotional stimuli (Kanske & Kotz, 2011c) or positive emotional stimuli (Kanske & Kotz, 2011a). These earlier studies reported shorter RTs for incongruent emotional than neutral trials, suggesting that negative and positive emotion both facilitate conflict processing. The divergent findings likely reflect differences in the task-context. While previous studies examined the effects of negative and positive emotional stimuli in separate sessions (Kanske & Kotz, 2011a, 2011c), we presented them in close temporal proximity in a fully randomized order within a run. In the present study, neutral, negative, and positive trials were presented in equal numbers within each experimental run to balance contrasts for positive and negative emotional stimuli. However, this resulted in greater potential for switching costs between trials of different congruence, arousal, and valence. This might have created an experimental context wherein higher cognitive effort was required to sustain attentional control. Furthermore, the switching between negative and positive emotional trials may have diluted their individual effect on conflict processing. Previous studies using neutral, negative, and positive emotional stimuli in a flanker task primarily focused on a carry-over effect of enhanced cognitive control that originated in the previous trial (Alguacil et al., 2013; Gratton et al., 1992;Landman & van Steenbergen, 2020 ; Zeng et al., 2016). More specifically, this means that the reaction time in the current trial is reduced if it is preceded by an incongruent compared with a congruent trial. While these studies (Landman & van Steenbergen, 2020; Zeng et al., 2016) reported increased engagement of cognitive control if the previous trial was incongruent for both negative and positive compared with neutral trials, they did not report any reduced conflict/interference effect for emotional compared to neutral conditions. This missing conflict effect for emotional stimuli is inconsistent with some previous studies (Kanske & Kotz, 2011a, 2011b, 2011c, 2011d, 2012b). This may be due to differences in experimental design (blocked or mixed) or the difficulty of the task (two-response vs. four response paradigms or two vs. four flankers). Considering that the influence of emotion on conflict processing may depend on the broader experimental context, future studies need to validate these interpretations by looking into the response switching costs and conflict adaptation, analyzing the effect of the previous (emotional/nonemotional or congruent/incongruent) trial on the reaction time or accuracy of the current trial (Chechko et al., 2014; Chen et al., 2009).

### Region of interest analysis: dorsal and ventral ACC

We replicated the expected increase in dACC activation for incongruent compared to congruent trials for neutral stimuli. However, with emotional stimuli the dACC also displayed increased activation for congruent trials (Fig. 3). Hence, while we confirm the expected role of the dACC in detecting a response conflict, we observed an unexpected increase in activation linked to emotion alone. This dACC response in emotional congruent trials may nonetheless be attributed to the presence of conflict or interference in these trials. While during the incongruent emotional trials, both the color of the top and bottom flanker words and the emotional meaning of the word interferes with the judgment of, and response to the task-relevant ink color of the centrally presented target word (Fig. 1), interference/conflict during congruent emotional trials is created only by the emotional meaning of the word, much like in the emotional Stroop trials (Feroz et al., 2019; Song et al., 2017). Accordingly, recruiting the dACC in both congruent and incongruent emotional trials can be attributed to its increased responsiveness to the occurrence of conflicts to the information processing (Botvinick et al., 2004; Mayer et al., 2012; Spunt et al., 2012; Xu et al., 2016). This interpretation is in line with previous conflict studies that report consistent dACC activity in the presence of conflict irrespective of the presence of emotional stimuli (Egner et al., 2008; Feroz et al., 2019; Kanske & Kotz, 2011c; Song et al., 2017; Spunt et al., 2012). Thus, the recruitment of dACC regardless of the level of conflict or emotionality of the stimulus, points toward a more general role of this brain region in assessing and monitoring incompatible information during parallel cognitive demands and appropriate response selection during conflict processing (Aarts et al., 2009; Brockett et al., 2020; Brockett & Roesch, 2021; Goldfarb & Henik, 2007; Mayer et al., 2012; Roelofs et al., 2006; Spunt et al., 2012; Xu et al., 2016).

According to previous studies (Kanske & Kotz, 2011c; Mohanty et al., 2007), activity in the vACC is associated with inhibition of emotional distractors and successful conflict resolution. This is depicted by faster responses during incongruent emotional trials or smaller conflict effect as compared to neutral trials or neutral conflict, respectively. Unlike these studies (Kanske & Kotz, 2011c; Mohanty et al., 2007), the constant high demand in attentional control that was likely introduced by all high-arousal emotional trials and/or the switching costs between trials may be the reason for the lack of a significant response in the emotional subdivision of ACC (Song et al., 2017).

### Whole brain neural activity

A fronto-parieto-cerebellar network of brain regions was more active during incongruent than congruent trials irrespective of stimulus type (Table 2). These results are in line with an extant literature that indicates the involvement of these regions in maintaining attentional/cognitive control in conflict processing irrespective of stimulus quality (e.g., emotionality). The right superior frontal gyrus is associated with conflict anticipation and inhibition of impulsive responses during conflict processing (Aarts et al., 2009; Hu et al., 2016; Ovaysikia et al., 2011), the middle frontal gyrus with inhibitory control and conflict processing in the presence of both emotional and nonemotional stimuli (Berron et al., 2015; Cservenka et al., 2015; Fan et al., 2007; Sebastian et al., 2017) and the middle/anterior cingulate cortex with conflict monitoring and detection regardless of the stimulus quality (e.g., emotionality) (Botvinick, 2007; Braem et al., 2017; Carter & van Veen, 2007; Kanske & Kotz, 2011c; Kim et al., 2013; Palermo et al., 2018). Similarly, the superior parietal lobule has been associated with response conflict and anticipation, contextual interference and biasing of attention (Berron et al., 2015; Durston et al., 2003; Fan et al., 2007; Fruhholz et al., 2009; Fruhholz et al., 2011) and the cerebellum with mediating conflict resolution by modulating response selection and biasing attention to detect change in the environment (Becerril & Barch, 2013; Kotz et al., 2014; Schweizer et al., 2007).

While both negative and positive emotion elicited a response in the left supramarginal gyrus, negative emotion activated a more extensive network of brain regions comprising the left and right middle temporal gyrus, the left middle temporal pole, the left superior/medial frontal gyrus, left angular gyrus/the left superior temporal gyrus, the left middle frontal gyrus, and the left inferior (orbital) frontal gyrus (Table 1). These brain regions have been suggested to play a role in controlling the interference caused by emotion in conflict processing. The supramarginal gyrus has been associated with emotion regulation and attention during conflict (Jiang et al., 2020; Olk et al., 2015; Wadden et al., 2018), the inferior frontal gyrus with emotion regulation and suppression (Beauregard et al., 2001; Berron et al., 2015; Egner, 2011; Kotz et al., 2015; Ochsner et al., 2004; Wittfoth et al., 2010), and the middle temporal lobes with developing stimulus specific representations and flexible relational rules (Dougal et al., 2007; Rose et al., 2002). These brain regions are associated with processes related to top-down emotion regulation and attention modulation rather than bottom-up emotion perception. This might imply that emotional stimuli in the current paradigm were engaging these brain regions to sustain and regulate attentional control to focus on task-relevant aspects and maintain task performance. Similarly, the lack of activation in emotion-specific brain regions such as amygdala in emotional trials, usually reported in conflict processing tasks (Cromheeke & Mueller, 2014; Kanske & Kotz, 2011c), also points to a dampening of bottom-up emotional reactivity to sustain attentional control and maintain task-performance (McRae et al., 2010).

These results indicate that emotional contexts are salient and influence appropriate response selection even when this selection is relatively straightforward (congruent emotional trials). Further research is needed to test whether the context produced by the temporal succession of emotional valences within either a fully randomized or blocked design systematically modulates response conflict. This mixing of opposite valence dimensions may be the reason why our findings did not disentangle how valenced stimuli influence conflict processing.

### Limitations

Some limitations and caveats should be noted. A potential limitation of the current study is the low number of trials per condition. This might have influenced that prior results could not be replicated (Kanske & Kotz, 2011b, 2011c, 2012b), in particular the significant interaction of emotion and congruence. Furthermore, in the view of the current sample size (N = 21), the current study might be underpowered to estimate valence-specific effects of emotion on conflict processing. However, previous studies with similar sample size (N = 20 to 26 in Kanske & Kotz, 2011a, 2011b, 2011c, 2011d) have reported significant interactions of emotion and congruence.

## Conclusions

This fMRI study sought to elucidate the influence of negative and positive emotion on conflict processing using a modified version of the Eriksen flanker task. Slower responses were observed for incongruent than congruent trials. However, no significant differences between negative and positive stimuli on conflict processing were observed. Functional MRI results pointed to a general role of dorsal ACC in monitoring and assessing conflict, as well as in selecting appropriate responses. Furthermore, the fMRI results showed that emotion enhances salience and drives appropriate response selection, even during low conflict, to accomplish task goals. Switching between trials of different congruence, arousal and valence may have created an experimental context that required higher cognitive effort to sustain attentional control. This also may have diluted the valence specific effects on conflict processing. Overall, our findings demonstrate that attentional control may help reduce the influence of emotional contexts in both high and low conflict situations to achieve overall task goals.
